# Cannabinoid Profiles in Medical Cannabis Users: Effects of Age, Gender, Symptoms, and Duration of Use

**DOI:** 10.1089/can.2020.0120

**Published:** 2022-12-05

**Authors:** Maja Kalaba, Mark A. Ware

**Affiliations:** Canopy Growth Corporation, Smiths Falls, Ontario, Canada.

**Keywords:** medical cannabis, real-world data, symptom management, pain, self-titration

## Abstract

**Introduction:**

Clinical trials remain the gold standard for evaluating efficacy, but there is increasing interest in using real-world evidence (RWE) to inform health care decision making. The aims of this observational study were to describe patterns of medical cannabis use, associated changes in symptom severity over time, and to evaluate change in cannabis dose over time for pain-related symptoms.

**Methods:**

Data were collected by Strainprint^™^, an application that is HIPAA, PIPEDA, and PHIPA compliant. A total of 629 participants recorded data between May 2017 and August 2019. A total of 65 symptoms were grouped as Pain, Mental Health, Physical Symptoms, Seizures, Headaches/Migraines, and Other. Descriptive statistics and mixed-effects modeling were applied.

**Results:**

THC-dominant products were more frequently consumed for symptoms of pain and sleep, while CBD-dominant products were more frequently consumed for anxiety and depression. Male and female participants demonstrated significant differences in the type of cannabis they consumed. Females more frequently consumed CBD-dominant products, and males more frequently consumed balanced (THC:CBD) products. Oil use was more prominent among females, while vaping was more common among males. Product use also varied by age tertiles (<31; 31–39; >40 years). CBD-dominant products were more common among younger participants, <31 years, THC-dominant products were more common among the 31–39 years category and balanced (THC:CBD) products were common among older participants >41 years. Dosages of CBD-dominant and balanced (THC:CBD) products increased over time irrespective of symptom response. THC-dominant products demonstrated a significant relationship between dose and symptom reduction over time.

**Conclusions:**

Recognizing that RWE has important methodological limitations, we observed cannabis product preferences based on demographic characteristics, such as gender and age and the primary symptom treated such as pain and anxiety. Our study offers real-world insights into how participants use and respond to cannabis products and suggests important avenues and methodologies for future research.

## Introduction

The pharmacologically active compounds in cannabis (cannabinoids) are known to moderate a range of physiological processes.^1–3^ The two main cannabinoids are THC and CBD. THC has been used as an analgesic agent, and is the major psychoactive ingredient in cannabis, whereas the nonintoxicating CBD has anxiolytic and anticonvulsant effects.^4,5^ THC administration has been shown to improve neuropathic pain,^6^ whereas CBD has been associated with improvements in anxiety and other neuropsychiatric disorders.^7,8^

Several studies have observed that medical cannabis may have therapeutic benefit in patients with headache (including migraine), gastrointestinal pain, arthritis, and joint stiffness.^3,6,9–15^ However, randomized controlled trials (RCTs) are often too small and too short to draw definitive conclusions.^16^ In addition, studies use different cannabinoid preparations and modes of administration, making meta-analyses complicated.^17,18^ In the last few years, more than 300,000 Canadians have received an authorization for medical cannabis as part of their treatment plan,^19^ despite the absence of RCTs that would inform clinical use. Understanding real-world utilization of cannabis (e.g., product selection, treatment intent, reported dose used, side effect profile, etc.) may generate evidence on safety and effectiveness and inform future clinical trial designs.

The utilization of real-world evidence (RWE) is becoming accepted as an independent research approach complementary to RCTs. RWE is defined as clinical evidence about the usage and potential benefits or risk of a medical product derived from real-world data (e.g., observational studies, registries, health record audits, data from wearables, etc.).^16,20^ There are several challenges with real-world data, including limitations of the data sources used and difficulties identifying and applying appropriate analytical tools to extract and interpret relevant information.^21^ Mobile applications (apps) are a form of innovative data collection, particularly for patient-reported outcomes.^21,22^ Development of these apps has led to increasing accessibility to large real-world datasets.

Given the patient-driven basis of medical cannabis authorization, and the wide range of potential therapeutic uses, RWE offers an important option to gathering insights into medical cannabis use patterns. To inform research programs and patient perspectives, we conducted an observational study using archival data from a mobile app to describe:

(1) the characteristics of participants as a function of the symptom being treated and product selection;
(2) the association of medical cannabis on self-reported symptom severity over time; and
(3) to examine self-titration patterns over time and determine how these patterns differ as a function of product profile (THC-dominant, CBD-dominant, and balanced) among participants treating pain symptoms.

## Methods

Data were collected using Strainprint^™^, a mobile app that is HIPAA, PIPEDA, and PHIPA compliant. Strainprint^™^ helps individuals track their medical cannabis use by monitoring therapeutic regimens (product, administration method, dose), and self-reported symptom scores. We examined Strainprint^™^ data collected prospectively from Canadian adults who had received a medical authorization to self-administer cannabis between May 2017 and August 2019. We included participants using data from one licensed producer (Spectrum Therapeutics) to standardize the exposure to products with known cannabinoid ratios.

**FIG. 1.**
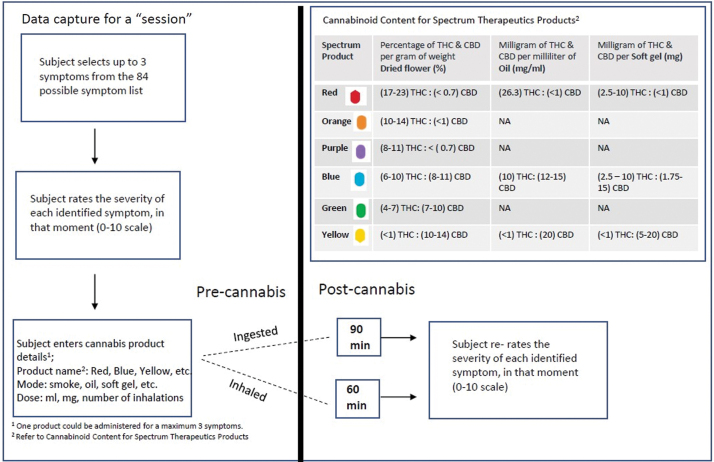
App session data capture. Process by which participants enter data on the app. App, application. Color images are available online.

Cannabis products are categorized based on cannabinoid content, reported as THC and CBD levels. Dried flower cannabis is reported in percent THC and CBD per gram weight of dried flower (%); soft gels are reported in milligrams of THC and CBD (mg) per capsule; and oils are reported as milligrams of THC and CBD per milliliter of oil (mg/mL) (Fig. 1). The Strainprint^™^ app recorded cannabis variables by product name, cannabis type (dried flower, oil, soft gel), mode of administration (inhaled or ingested), and dosage (in “puffs” for inhalation, mg for soft gel, and mL for oils) (Fig. 1). Due to the limitations of using puffs as a dose measure (e.g., differences in puff volume, variations in breath-holding techniques, combusting vs. vaporization), self-titration patterns were estimated using dosage as an outcome for ingested products only, reported in mL per day. Soft gel (mg) was converted into mL using the concentration indicated on the product label (e.g., a single 2.5 mg red soft gel was treated as the equivalent of 0.1 mL of red oil 26.3 mg/mL).

Participants recorded data at several “sessions” defined as data entry on the app. During the initial session, participants recorded their demographics, including age, gender, province of residence, and medical conditions. Participants self-reported their symptoms and described their medical cannabis treatment regimen. Symptoms were identified from a list of 84 symptoms on the app. For the purposes of this study, symptoms were divided into 6 groups: Pain, Mental Health, Physical Symptoms, Seizures, Headaches/Migraines, and Other (Table 1).

**Table 1.** Grouping of Selected Strainprint^™^ Symptoms (*n*=65)

Pain (*n*=10)	Mental health (*n*=12)	Physical symptoms (*n*=16)	Headaches/migraines (*n*=3)	Seizures (*n*=3)	Other (*n*=21)
Arm or leg pain Back pain-lower Back pain-upper Breakthrough pain Joint pain Knee pain Muscle pain Neck pain Nerve pain Sciatica	Anxiety Challenge concentrating Compulsive behavior Depression Hopelessness Intrusive thoughts Irritability Panic attacks Paranoia PTSD-flashbacks Repetitive behavior Stress	Chest pain Cramps Diarrhea Difficulty breathing Dizziness Gastrointestinal pain Itchiness Joint stiffness Nausea Numbness/tingling Paresthesia Restlessness Rigidity Sweating Tenderness Weakness	Headaches Migraine with aura Migraines	Absence of seizures Focal seizures Seizures	Dental pain Eye pressure Fatigue General discomfort Inflammation Insomnia Lack of appetite Light sensitivity Low libido Muscle spasms Pelvic pain PMS Rash Recreational Skin sensitivity Spasticity Temporary partial paralysis Tinnitus Touch sensitivity Tremor Withdrawal

PMS, premenstrual syndrome; PTSD, post-traumatic stress disorder.

Each session captured data before and after cannabis use. In the pre-cannabis use phase, participants identified up to three symptoms that they intended to treat with medical cannabis. The severity of each symptom was rated on an 11-point numerical rating scale (0: lowest severity; 10: highest). In the post-cannabis phase, participants entered data after cannabis use. Participants who inhaled cannabis were prompted 60 min post-administration, whereas those who ingested cannabis orally were prompted 90 min after administration (this was standard procedure for Strainprint^™^ users at the time of the study). These intervals are consistent with the known pharmacodynamic differences between these routes of administration.^23^ Following the prompt, participants rated symptom severity using the same 11-point scale (Fig. 1). We received de-identified data from Strainprint^™^ to conduct our analysis. All individuals who register on the App signed a Consent to Collection and Use of Data form for research purposes. The data collection methods for the current analysis are similar to those used in previously published research using this app.^12,24^ The data collected were used for three analyses.

### Analyses to address objective 1

To describe the characteristics of participants as a function of the symptom being treated and product selection descriptive statistics were generated across participants, and between groups of symptom and cannabis product. The data were stratified by gender and age tertiles (<31; 31–39; >40 years). Summary statistics included the mean and standard deviation (SD) for continuous variables and counts and percentages for categorical variables.

### Analyses to address objective 2

The aim of this analysis was to describe associated changes in symptom severity over time. Data were included for Analysis 2 if: (1) participants completed at least two sessions through Strainprint^™^, and (2) participants self-administered a cannabis product that was THC-dominant (Red, Orange or Purple), CBD-dominant (Yellow, Green), or balanced (Blue).

Change in symptom severity from pre- to post-cannabis use was assessed with the paired-samples *t*-test. Due to considerable variability in the number of recorded sessions by participants, we made comparisons between the mean baseline measurement (first session pre-cannabis symptom severity score), and three post-cannabis symptom severity scores grouped as follows:

(1) the mean post-cannabis symptom severity score derived from the first session,
(2) the mean post-cannabis symptom severity score derived from the last session, and
(3) the mean post-cannabis symptom severity score showing greatest reduction.

To determine whether there were any differences between symptom improvement and product used, three one-way between-group analyses of variance (ANOVAs) were run. These analyses used each of the aforementioned outcome variables (e.g., the mean post-cannabis symptom severity score derived from the first session), with product used (Red, Orange, Purple, Yellow, Green, and Blue) entered as the grouping variable. To control for alpha rate inflation due to multiple comparisons, we Bonferroni corrected the alpha rate to 0.017 for three comparisons for the *t*-test and ANOVA analyses. Descriptive and inferential statistics were calculated using IBM SPSS^®^ Statistics 24.^25^

### Analyses to address objective 3

**FIG. 2.**
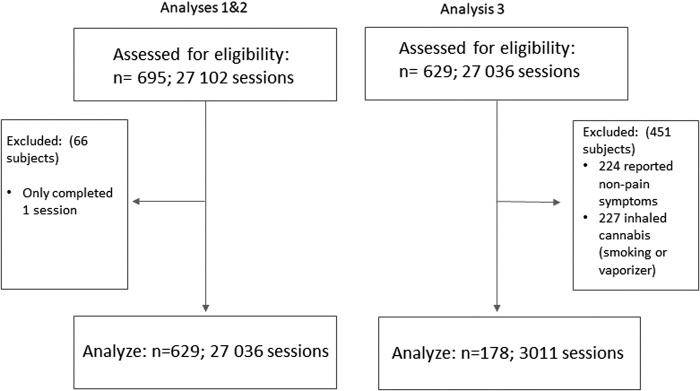
CONSORT flow diagram. Participants assessed for eligibility, inclusion and exclusion for first, second, and third analyses.

The aim of this analysis was to examine self-titration patterns over time, and to determine how these patterns differ as a function of product profile among participants treating pain symptoms. Data were included for Analysis 3 if (1) participants indicated a symptom of pain, and (2) participants who exclusively ingested cannabis as an oil or soft gel that was THC-dominant (Red), CBD-dominant (Yellow), or balanced (Blue) (Fig. 2). Data capture on inhaled cannabis was excluded from this analysis, due to our concerns about the validity of the number of puffs representing a dose measure. When a patient inhales cannabis, there are several variables that impact the actual amount consumed (e.g., total grams, length of inhalation, etc.), which were not captured on the app. Only the first 10 sessions were included in analyses owing to substantial dropout after the 10th session.

An effectiveness score was defined as the within-participant change in pain at each session. Effectiveness score was calculated as post-cannabis use symptom severity score − pre-cannabis use symptom severity score for each participant. As such, negative effectiveness scores reflected a reduction in symptom severity.

To evaluate dose change over time, linear mixed-effects modeling was undertaken. These models have the advantage of using all available data to estimate parameters and allow for inclusion of cases with missing data under the missing-at-random assumption.^26^ Models included random intercepts and slopes to account for the correlated nature of the data within participant over time. Fixed effects of product, time (session), and effectiveness score were included, and all possible interactions among these variables. Nonsignificant higher-order interactions were removed from models to achieve maximal model fit. Relative model fit was evaluated using likelihood ratio tests to confirm model selection. Sequential instance of app use (sessions) was used as the time metric, which was treated as a continuous repeated measure. The CBD-dominant product was the reference category for the product predictor, and effectiveness score was mean centered. Owing to substantial positive skew, dose was log transformed. All parameter estimates are presented in the log-transformed scale in the text, but were back transformed to reflect predictor effects on the original dose scale in figures, to facilitate interpretation. Restricted maximum likelihood estimation was used to estimate and test individual parameters and full maximum likelihood estimation was implemented for evaluation of relative model fit. The “nlme” package in R version 3.6.1 was used for mixed-effects modeling.^27^

## Results

### Participant characteristics (objective 1)

A total of 629 unique Spectrum product users who collectively entered 27,036 sessions over the 27-month period were included in Analysis 1. The participants' mean age was 38.3 years ±10.7 (range 18–74 years), and (58.7%) identified as female. The majority of users resided in Canada (97.1%), with the largest proportion from Ontario and Alberta (40% and 31%, respectively). The median number of medical conditions identified per individual was 4, with anxiety disorder, back pain, and chronic pain as the most frequent (Table 2). The number of months over which participants recorded data on Strainprint^™^ (time from baseline to last session) ranged from 5 to 12 months.

**Table 2.** Characteristics of Application Participants (Recorded at Initial Session)

Characteristics	*N* (%)
No. of participants	629
Mean±SD age (years)	38.3±10.8
Age range (years)	18–74
Female	(58.7)
Province of residence	540
Ontario	212 (40)
Alberta	166 (31)
British Columbia	41 (8)
Quebec	34 (6)
Manitoba	33 (6)
Newfoundland and Labrador	12 (2)
New Brunswick	9 (2)
Nova Scotia	5 (1)
Saskatchewan	3 (0.5)
Prince Edward Island	2 (0.3)
Unknown	23 (4)
Missing data	89
Conditions^a^	
Mean±SD	6.6±7.6
Median	4
Minimum	1
Maximum	12
Conditions^a^ (>50 participants identified the condition)	
Anxiety disorders	321
Back pain	236
Depression	221
Stress disorder	179
Insomnia	172
Chronic pain	138
Arthritis	116
Lower back pain	112
Headaches, migraines	104
Pain—general	101
Headaches, tension	98
Migraines	78
PTSD	74
IBS	72
Fibromyalgia	64
Sleep disorders	60
Any chronic medical condition that limits major life activities	57
Attention deficit disorder	51
Knee, ankle, or foot injury	50

^a^ Conditions were captured as a “select all that apply” variable. Included in the table are the most frequently selected conditions identified by a minimum of 50 participants.

IBS, irritable bowel syndrome; PTSD, post-traumatic stress disorder; SD, standard deviation.

**FIG. 3.**
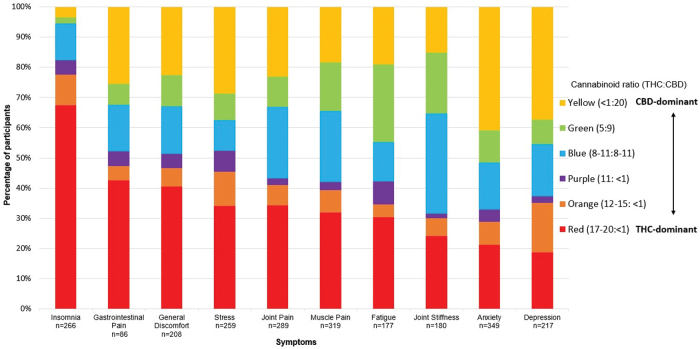
Product utilization by symptom (*n*=629). Participant self-reported Spectrum Therapeutics product consumed by symptom. Symptoms displayed: Insomnia, Gastrointestinal Pain, General Discomfort, Stress, Joint Pain, Muscle Pain, Fatigue, Joint Stiffness, Anxiety, Depression. Note: Participants could select up to three symptoms per session. Color images are available online.

The first analysis describes the characteristics of participants who used Strainprint^™^ to record their therapeutic use of cannabis. In total, 65 symptoms were identified across the 629 participants. The most commonly treated symptoms were muscle pain (10.7%), anxiety (10.1%), joint pain (9.2%), insomnia (7.4%), and joint stiffness (6.8%). Insights on product preferences across symptoms are shown in Figure 3.

Male and female participants demonstrated significant differences in the type of cannabis they consumed (Table 3).

**Table 3.** Cannabis Consumption, Significant Differences by Gender and Age

Consumption of	Gender			χ^2^ (*p*-value)
	Female (%)	Male (%)		
Ingested oils	53.8	30.2		4.5 (0.001)
Dried flower (Vaping)	25.7	39.9		
Balanced product (THC:CBD)	17.5	22.0		2.2 (0.022)
CBD-dominant product	29.4	19.0		
THC-dominant product	18.2	24.0		

	**Age Tertiles**			
	**<31 Years (%)**	**31–39 Years (%)**	**>40 Years (%)**	
Balanced product (THC:CBD)	10.1	12.7	30.8	6.9 (0.001)
CBD-dominant product	32.5	24.4	18.7	
THC-dominant product	27.7	40.4	29.4	

### Symptom severity scores over time (objective 2)

**FIG. 4.**
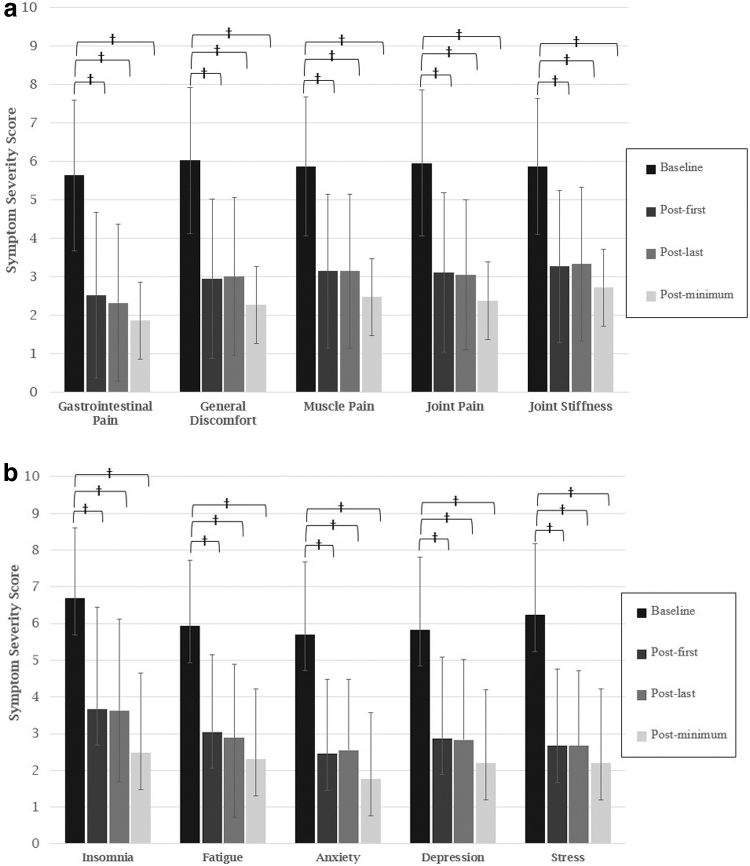
**(a)** Change in symptom severity scores over time for physical and pain symptoms. Note: Mean time elapsed from baseline to last session in months (range). Gastrointestinal Pain: 7.9 (7.3–8.6). General Discomfort: 8.4 (8.1–9.2). Muscle Pain: 9.8 (9.5–10.1). Joint Pain: 9.8 (9.5–10.1). Joint Stiffness: 12 (11.7–12.3). Error bars reflect 95% confidence intervals. ^†^*p*<0.001. **(b)** Change in symptom severity scores over time for mental health and other symptoms. Note. Mean time elapsed from baseline to last session in months (range). Insomnia: 8.2 (7.9–8.5). Fatigue: 9.0 (8.7–9.4). Anxiety: 8.4 (8.2–8.7). Depression: 10.3 (9.9–10.7). Stress: 5.9 (5.5–6.4). Error bars reflect 95% confidence intervals. ^†^*p*<0.001.

Figure 4a and b portrayed that in paired analysis comparing all symptoms across the three timepoints, to the respective baseline score, clinically meaningful and statistically significant improvements were observed (all *p*s<0.001). For Physical and Pain symptoms, all scores improved by a minimum of 2.6 points relative to baseline score. For Mental Health and Other symptoms, all scores improved by a minimum of 2.8 points relative to baseline score.

Results of the one-way ANOVA revealed no significant differences between selected product in terms of symptom improvement (all *p*s>0.017).

### Self-titration patterns over time (objective 3)

Of the 405 participants who reported pain symptoms, 178 (44%) ingested cannabis (oil or soft gel) over 3011 sessions and were included in the analysis. Participants' mean age was 40.8 years (range 18–74 years), evenly balanced by gender (52.8% male). The median number of days to complete 10 sessions was 16 (interquartile range: 8–40).

**FIG. 5.**
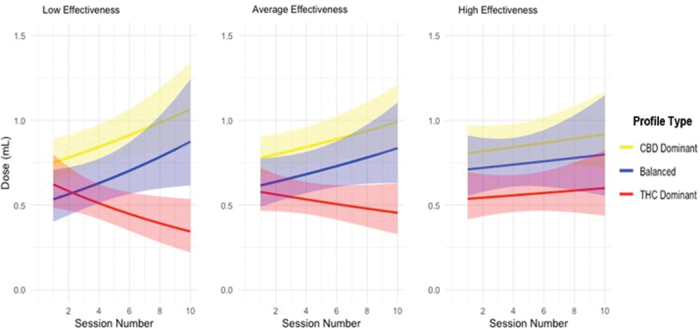
Model-predicted dose change trajectories across app use sessions by profile type (CBD-dominant, Balanced (THC:CBD), THC-dominant). Note. Low effectiveness score=0.41-point symptom reduction; average effectiveness score=2.54-point symptom reduction; high effectiveness score=4.67-point symptom reduction. Color images are available online.

Evaluation of relative model fit indicated that the highest order model, including the three-way (time×product×effectiveness score) interaction yielded better fit than all other lower order models (all *p*s<0.05). The model-predicted trajectories of dose change over time for each product, across all 10 sessions at levels of low (−0.41), average (−2.54), and high effectiveness score (−4.67) (Fig. 5). At session 1, there were no differences in dose (mL) across products (all *p*s>0.066).

For the CBD-dominant product users, an increase in dose across sessions was observed (*b*=0.03, *t*=2.11, *p*=0.035), and balanced product users followed the same trajectory of dose increase over time (i.e., there was no difference between CBD-dominant product users and balanced product users; [*b*=0.01, *t*=0.332, *p*=0.740]). In contrast, THC-dominant product users showed less of an increase in dose over time relative to the CBD-dominant product users, and in fact showed a decrease in trajectory (*b*=−0.05, *t*=−2.164, *p*=0.031). However, this effect was qualified by a higher-order three-way THC dominant×effectiveness×time interaction (*b*=−0.02, *t*=−2.759, *p*=0.006). High effectiveness (1 SD above average effectiveness) did not correspond to a significant increase in dose either for THC-dominant users (*b*=0.01, *t*=0.53, *p*=0.60), or CBD-dominant users (*b*=0.01, *t*=0.86, *p*=0.39). Average levels of effectiveness corresponded with a nonsignificant decrease in dose for THC-dominant users (*b*=−0.03, *t*=−1.18, *p*=0.24) and an increase in dose for CBD-dominant users (*b*=0.03, *t*=2.14, *p*=0.03). Low effectiveness (1 SD below average effectiveness) corresponded with a significant decrease in dose for THC-dominant users (*b*=−0.07, *t*=−2.12*, p*=0.03) and increase in dose for CBD-dominant users (*b*=0.04, *t*=2.57, *p*=0.01) (Fig. 5).

At average level of effectiveness score for CBD-dominant and balanced product, the models predicted trajectories suggesting an increase in dose across the 10 sessions of 0.21 and 0.22 mL, respectively. For the THC-dominant product, at low and average level of effectiveness score, there was a decrease in dose across the 10 sessions of 0.28, and 0.12 mL, respectively, and at high levels of effectiveness score, there was an increase in dose across the 10 sessions of 0.10 mL.

## Discussion

We completed an observational analysis of prospectively collected archival data on patterns of medical cannabis use and self-reported symptom reduction in participants with a variety of conditions.

We observed that THC-dominant product use was frequently selected for the treatment of sleep and pain (gastrointestinal pain in particular), while CBD-dominant product use was frequently selected for the management of mental health conditions, such as anxiety and depression. The current study is unable to determine how products were selected. It will therefore be important for future work to examine if physician, patient preferences or a combination of the two drives product selection. Product and administration differences were also observed by age and gender. These observations suggest that there are subsamples of the population, such as age and gender that differ in product selected. For example, CBD-dominant products were more common among younger participants (<31 years of age), THC-dominant products were more common among the 31–39 years category, and balanced (THC:CBD) products were common among older participants (>41 years).

While our analyses show a significant reduction in symptom severity over time among participants, we first acknowledge that there are considerable limitations in interpreting these findings. Positive effects were observed across all cannabis types and symptoms. Such a robust response to a complex array of cannabinoids on a range of symptoms is difficult to interpret based on classical single-agent pharmacology. Indeed, recent reviews conclude that there, is at best, weak evidence for cannabinoids being effective in managing specific symptoms.^28,29^ One possible explanation for these observations therefore would be the presence of a significant and prolonged placebo effect. Patients with chronic diseases may have a high degree of expectation and this may effect a significant bias on the results. While there is emerging scientific rationale to suggest responses of anxiety disorders to CBD^30–32^ and pain to THC,^11^ robust large-scale trials are lacking, and we are only just beginning to explore complex patterns of poly-cannabinoid use for complex chronic disorders. Clearly there is a need for additional focused trials to examine cause and effect relationships, but in addition, efforts are needed to identify appropriate control groups for large well-phenotyped observational studies to better explore the relationship between medical cannabis use and therapeutic response in real-world settings.

There are other limitations to consider. Stemming from the use of real-world data, the design of this analysis lacks a control group. Evidently, there is a sampling bias, which overrepresents participants who find medical cannabis effective, since participants who do not find medical cannabis effective would be unlikely to continue to use Strainprint^™^. A total of 66 (9.5%) participants only completed one session on the app. The unit of puff for inhalations lacks the necessary detail (e.g., grams consumed, length of inhalation, duration of breath hold, temperature setting of devices, etc.) for granular analyses of these data, limiting our self-titration results to oils and soft gels. In addition, there was considerable attrition in the sample (e.g., significant dropout after the 10th session), which may have biased parameter estimates. Overall, while we have a large and unique dataset to explore, loss to follow-up and the limitations of self-reported use data restrict the conclusions we can draw from the analyses completed. Tolerance to therapeutic effect and tolerance to adverse effects may have impacted treatment outcomes. As such, we cannot advance conclusions as to the causes of the effects observed here. Delineating the underpinning mechanism of the dosing changes found in this study is an area of future research.

Notwithstanding the limitations outlined above, what lessons can we take away? The data were obtained from authorized medical cannabis users, which required a physician approval. Furthermore, a wide variety of products were used by a large sample to treat different symptoms in a real-world setting. These results may therefore have ecological validity and represent how medical cannabis is being used in everyday life. Symptomatic improvements were described as early as the first session, which suggest that subjects self-report a quick onset of symptom relief with the initial cannabis dosage. Moreover, reductions in symptom severity up to 12 months from their first session were noted in participants with longer follow-up data, suggesting that, for these patients, therapeutic benefit was sustained over time.

Our findings on patient preference are consistent with others; Li et al.^6^ found that THC-dominant products, as compared with CBD-dominant, were more frequently selected in treating pain. If THC-dominant products are effective analgesics, it would explain why we observed a tendency for participants to select THC-dominant products in the treatment of pain. Gulbransen et al.^8^ observed that CBD-dominant cannabis was associated with improvements in pain, anxiety, and depression, in patients with noncancer pain and mental health symptoms, although their study design also precludes stronger inferences on correlation. Black et al.^33^, points out the scarce evidence for cannabinoids in improving depressive disorders and symptoms, requiring further high-quality studies. While CBD has been found to be relatively safe,^34^ it is not risk free, and potential drug/drug interactions must be considered when recommending it to patients.^35^

Our data on dosage of cannabis and changes in dosage over time are also consistent with other studies.^12,36^ We found overall increases in dosage and effectiveness score over time for participants treating pain symptoms. Changes in cannabis dosage over time varied by effectiveness score, suggesting that participants titrated dosages based on perceived effectiveness. This effect differed by the cannabinoid content of the cannabis consumed. This may be interpreted as reward-seeking behavior: by increasing the amount of cannabis consumed, participants aim to enhance previous effectiveness. The observation may also be an effect of tolerance, whereby increasing dosages are required to yield a satisfactory effectiveness.

When the effectiveness in reducing symptoms was low, dosages of THC-dominant product decreased, whereas dosages of CBD-dominant and balanced increased over time. This effect may be explained as participants' aversion to the effects of THC-dominant products found to be ineffective at symptom reduction, discouraging an increase in dosage. However, as CBD-dominant and balanced products would not be expected to give rise to the same psychoactive experiences as high dosages of THC products, participants may have been more comfortable to increase the dosage of the former to improve effectiveness.

Analyses of real-world data on medical cannabis use may reveal patterns of use and effectiveness across individuals in natural conditions that could inform treatment decisions and stimulate future research. Improvements in app design and compliance would conceivably increase the quality of RWE available in the future. Safety monitoring plays a crucial role in understanding various aspects of usage patterns (compliance, titration, duration of treatment). Systematic collection of adverse events on apps would further strengthen future RWE analyses.

## Conclusions

This study provides real-world insights into patterns of use for medical cannabis, self-titration trends, and responses to various cannabis products within the first month of initiating treatment for different symptoms. While recognizing that RWE studies have limitations, the study builds upon evidence of safety, efficacy, pharmacokinetics, and therapeutic indications for medical cannabis. In this study, we specifically observed product preferences based on the symptoms treated (e.g., CBD-dominant products for symptoms of anxiety and depression) and demographic characteristics (e.g., CBD-dominant use was more frequent in females). An early onset of symptom improvement was noted with cannabis use, and it was sustained over time. Upon first use of medical cannabis oil for pain symptoms, participants reported pain reduction irrespective of the dose and product used. Only THC-dominant products demonstrated a statistically significant relationship between dose and effectiveness score over time. In the ongoing quest for producing the best evidence-based medicine, we suggest that both RCT and RWE data hold unique value and complement each other.

## Abbreviations Used

ANOVAs: analyses of variance
app: application
CBD: cannabidiol
IBS: irritable bowel syndrome
PMS: premenstrual syndrome
PTSD: post-traumatic stress disorder
RCT: randomized controlled trial
RWE: real-world evidence
SD: standard deviation
THC: tetrahydrocannabinol
